# *Leopard-EM*: an extensible 2D template-matching package to accelerate *in situ* structural biology

**DOI:** 10.1107/S2059798325009982

**Published:** 2026-01-01

**Authors:** Matthew D. Giammar, Joshua L. Dickerson, Laina N. Hall, Bronwyn A. Lucas

**Affiliations:** a Center for Computational Biology, University of California Berkeley, Berkeley, California, USA; b Department of Molecular and Cell Biology, University of California Berkeley, Berkeley, California, USA; c California Institute for Quantitative Biosciences (QB3) University of California Berkeley California USA; d Biophysics Graduate Program University of California Berkeley California USA; e Molecular Biophysics and Integrated Bio-Imaging, Lawrence Berkeley National Laboratory, Berkeley, California, USA; University of Cambridge, United Kingdom

**Keywords:** *in situ* structural biology, cryo-EM, cryo-ET, template matching, structural cell biology

## Abstract

The ability to generate high-resolution views of cells with cryogenic electron microscopy (cryo-EM) can reveal the molecular mechanisms of biological processes in their native cellular context. The revolutionary impact of this strategy is limited by the difficulty of accurately annotating structures within these images. 2D template matching (2DTM), in which high-resolution structural models are used as computational probes to locate and orient molecular complexes with high precision, has shown initial promise in annotating single molecules in cellular cryo-EM images. While the scientific community works to identify best practices for applying 2DTM to specific biological questions and to maximize sensitivity and throughput, a modular and extensible software architecture would support the rapid development of novel methodological approaches, thus accelerating innovation within the field. To achieve this, we developed *Leopard-EM* (***L**ocation and ori**e**ntati**o**n of **par**ticles found using two-**d**imensional t**E**mplate **M**atching*), a modular Python-based 2DTM implementation built to be easily customizable. We implemented an automated pixel-size refinement procedure and find that 2DTM is sensitive to pixel-size errors of ∼0.2%. To demonstrate the flexibility of the *Leopard-EM* architecture, we developed a constrained search protocol that improved small ribosomal subunit detection by approximately eightfold by using initial locations and orientations determined for the large ribosomal subunit. Using this strategy, we captured a distribution of ribosome rotation states within the living cell at single-molecule resolution. We envision that *Leopard-EM* can be used as a platform for the development of *in situ* cryo-EM data-processing workflows, facilitating the rapid development of this field. *Leopard-EM* is available at https://github.com/Lucaslab-Berkeley/Leopard-EM.

## Abstract 2

*In situ* cryo-EM coupled with 2D template matching (2DTM) holds the potential to visualize the cellular structureome in context, but further developments are required to make this a reality. We describe *Leopard-EM* (***L**ocation and ori**e**ntati**o**n of **par**ticles found using two-**d**imensional t**E**mplate **M**atching*), an extensible Python package for 2DTM to accelerate *in situ* structural biology.

## 1. Introduction

Cryogenic electron microscopy (cryo-EM) can generate high-resolution micrographs of cells, each containing a large number of biological macromolecules in their native cellular environment. Reliable annotation of specific biological macromolecules within these micrographs is the rate-limiting step of *in situ* structural biology. In contrast to single-particle analysis (SPA), in which particle-picking methods use high-contrast, low-resolution features, many cellular complexes are visually indistinguishable relative to the cellular milieu. Moreover, cell sections are typically 100–200 nm thick, resulting in a higher proportion of multiple and inelastic scattering, reducing the signal-to-noise ratio (SNR) relative to SPA. These limitations, combined with the inherently low SNR of cryo-EM images due to radiation damage and the low-dose collection scheme, make the specific annotation of even a handful of the ∼20 000 proteins in the proteome an extreme challenge.

Improved accuracy in structure prediction has made it possible to generate a reference ‘structureome’ from genomic sequences (Jumper *et al.*, 2021 ▸; Baek *et al.*, 2021 ▸; Tunyasuvunakool *et al.*, 2021 ▸; Lucas, 2023 ▸). The greatly increased number of available structural models has made template matching a viable strategy to locate proteins in cryo-EM images of cells. Template matching in cryo-EM refers to several strategies in which a reference is used to detect a particular feature of interest by cross-correlation with the data. Template-matching strategies vary in the type of template used, the inclusion and type of noise applied to the template, the number of alternative templates considered, the filters applied and the way in which the results are normalized. Each of these differences changes the interpretation of the results and makes a quantitative comparison challenging.

Even in crowded cellular environments, high-resolution structural features remain distinct and are best preserved in full-exposure images of untilted samples, rather than in tomograms (Dickerson & Lucas, 2025 ▸). Two-dimensional template matching (2DTM), by exploiting these high-resolution features, can be used to determine the location and orientation of macromolecules in cryo-EM images with high precision, even in the presence of background noise (Sigworth, 2004 ▸; Rickgauer *et al.*, 2017 ▸). In 2DTM, the presence of a particular complex is assessed by calculating the cross-correlation of spectrally whitened projections, corresponding to specific poses of a noise-free 3D reference template, with the whitened image. The cross-correlation of the best-matching orientation at each pixel is stored, as well as the rotational offset to generate the projection from the template. Matches are detected by thresholding based on the probability of a false detection given Gaussian noise and the total number of cross-correlations performed. 2DTM is, in essence, an implementation of the classical matched filter (Sigworth, 2004 ▸), which is the theoretically optimal linear filter for the detection of a signal of interest in the presence of additive random noise.

2DTM has been applied to identify the location and orientation of VP1 polymerase in rotavirus DLP particles (Rickgauer *et al.*, 2017 ▸) and ribosomes in thin extensions of mammalian cells (Rickgauer *et al.*, 2020 ▸, 2024 ▸), as well as ribosomes and ribosomal subunit precursors in *Mycoplasma pneumoniae* (Lucas *et al.*, 2021 ▸) and FIB-milled yeast lamellae (Lucas *et al.*, 2022 ▸). Related strategies have been applied to locate candidate particles of interest for subsequent high-resolution 3D reconstruction from 2D images (Cheng *et al.*, 2021 ▸; Lucas *et al.*, 2023 ▸), and have generated high-resolution *in situ* structures of translating ribosomes (Cheng *et al.*, 2025 ▸; Zheng *et al.*, 2024 ▸) and large photosynthetic supercomplexes (You *et al.*, 2023 ▸).

2DTM has the potential to visualize the proteome in context, but further improvements in sensitivity and throughput are required to achieve this goal. Moreover, customization is required to address specific biological questions or data sets. For example, comparing 2DTM *z*-scores from multiple templates was used to discriminate structurally related large ribosomal subunit (LSU) maturation intermediates within the yeast nucleus (Lucas *et al.*, 2022 ▸). In another example, mammalian translation dynamics were estimated by ordering ribosome–cofactor complexes on the basis of their conformational state (Rickgauer *et al.*, 2024 ▸). Alternate metrics to call significant detections have also been proposed (Zhang *et al.*, 2025 ▸), which may find applications to address specific biological questions. Several extensible software packages have been developed for 3D template matching (3DTM) in tomograms (Maurer *et al.*, 2024 ▸, 2025 ▸; Chaillet *et al.*, 2025 ▸), providing frameworks for flexible methodological development in that domain. However, comparable modular and customizable implementations for 2DTM are lacking.

Realizing the potential of 2DTM requires a modular and accessible software architecture that can easily be adapted by various users to fit the application at hand. Here, we describe a new modular implementation of 2DTM, *Leopard-EM*, which is written in Python, leveraging PyTorch for GPU acceleration. This tool was designed to be extensible for rapid development by a broad user and developer base. We show how *Leopard-EM* can be used in a standard 2DTM workflow and demonstrate the importance of screening template pixel size to maximize detection sensitivity. We demonstrate the extensibility of *Leopard-EM* by presenting a use case in which we constrain a local search for the small ribosomal subunit using results from a prior template-matching search for the large ribosomal subunit, enabling the identification of intersubunit rotation with single-molecule precision.

## 2. Methods

### 2.1. Yeast-cell culture, grid preparation and FIB milling

*Saccharomyces cerevisiae* strain W303 haploid (NucLoc) colonies were inoculated in 5 ml YPD medium and grown overnight at 30°C with shaking (200 rev min^−1^). Cultures in mid-log phase (∼0.8 OD) were selected and diluted to 10 000 cells ml^−1^. 3 µl were applied to a Quantifoil 2/2 SiO_2_ 200 mesh Cu grid, allowed to rest for 15 s, back-side blotted for 8 s at 27°C and 95% humidity, and plunge-frozen in liquid ethane at −184°C using a EM GP2 cryo-plunger (Leica). Frozen grids were stored in liquid nitrogen until FIB milling.

### 2.2. FIB milling

Lamella were generated using a Hydra cryo-FIB-SEM (Thermo Fisher) and a Xenon ion source operated at 30 kV. Lamella were prepared using *AutoTEM* (ThermoFisher) with the following protocols. Stress-relief cuts were milled at a current of 0.3 nA. Rough milling was performed at 0.3 nA. Medium milling was performed at 0.1 nA using a cleaning cross-section pattern, followed by finer milling at 60 pA, ultimately resulting in a targeted thickness of ∼500 nm. Polishing was performed at 6 pA to a target thickness of 200 nm. Electron images were not taken during lamella preparation.

### 2.3. Cryo-EM data collection and preprocessing

Cryo-EM data were collected following the protocol described in Lucas *et al.* (2022 ▸) using a ThermoFisher Krios 300 kV electron microscope equipped with a Falcon 4 camera and Selectris X energy filter at a nominal magnification of 81 000× (pixel size of 0.93 Å^2^) and a 100 µm objective aperture. Movies were collected to a total fluence of 50 e^−^ Å^−2^ with 1800 frames in EER mode targeting a defocus of 0.5 µm. Cytoplasm was targeted by selecting regions of interest in a low-magnification overview. The movie frames were aligned using *MotionCor*3 (Zheng *et al.*, 2017 ▸) and dose-weighted according to Grant & Grigorieff (2015 ▸). CTF and thickness estimation were performed using* CTFFIND*5 (Elferich *et al.*, 2024 ▸), which estimated a defocus range from 0.36 to 1.05 µm.

### 2.4. 2DTM search for the large ribosomal subunit and small ribosomal subunit body

In summary, we used a PDB model of the 80S ribosome in the nonrotated state, PDB entry 6q8y (Tesina *et al.*, 2019 ▸), and manually generated two additional PDB files, one from the large ribosomal subunit (LSU) alone and one from the small ribosomal subunit (SSU). The SSU was further processed by the removal of RACK1, RPS29A, RPS28A, RPS25A, RPS20, RPS12, RPS31, RPS3, RPS10A, RPS15, RPS18A, RPS19A, RPS5P, RPS16A, RPS0A, RPS17A and nucleotides 1128–1609 of the modeled 18S rRNA to generate the SSU ‘body’ template, which contains the body, platform and shoulder. The PDB files for the templates used can be found in the EMPIAR submission accompanying this paper. These models were aligned with the full model in *ChimeraX* (Pettersen *et al.*, 2021 ▸). The 80S model was re-centered at coordinates (0, 0, 0), and the same transformation was applied to the other models using a custom Python script. Using a pixel size of 0.936 Å, we simulated a 512 × 512 × 512 pixel model using the program *ttsim*3*d*, with the parameters of no re-centering, no additional *B*-factor and *B*-scaling of 0.5 relative to those deposited in the PDB. Only non-H atoms were included in the simulation. We ran *match_template* in *Leopard-EM* with uniform angular sampling, with a ψ step of 1.5° and a θ step of 2.5° with *C*1 symmetry. The CTF *B*-factor was 0 Å^2^. The defocus search range was −1200 to 1200 Å in 200 Å steps.

After running *match_template*, the peaks were extracted from the *z*-score map using a threshold of one false positive per micrograph. The resulting peaks were further refined, using a defocus search of −100 to 100 Å in 20 Å steps, an extracted box size of 518 × 518 pixels and angular steps of 0.05°. In total, we located 12 357 LSUs from 70 micrographs (Fig. 4*b*).

All scripts used to perform 2DTM can be found at https://github.com/Lucaslab-Berkeley/Leopard-EM_manuscript.

### 2.5. 2DTM search for proteasome

We used the PDB model of the 20S yeast proteasome, PDB entry 1ryp (Groll *et al.*, 1997 ▸), to simulate a 384 × 384 × 384 pixel model at 1.059 Å using the program *ttsim*3*d* with no *B*-factor scaling or additional *B*-factor applied to atoms. Only non-H atoms were included in the simulation. We ran *match_template* in *Leopard-EM* on 28 previously published micrographs (Lucas *et al.*, 2021 ▸) with uniform angular sampling using a ψ step of 1.5°, a θ step of 2.5° and *C*2 symmetry.

All scripts used to perform 2DTM can be found at https://github.com/Lucaslab-Berkeley/Leopard-EM_manuscript.

### 2.6. Constrained search for SSU

To search for the SSU, we simulated a 3D map of the SSU ‘body’ using the same parameters as for the LSU. We ran *match_template* in *Leopard-EM* using the same parameters and used the variance from this result for the *z*-score calculation in subsequent constrained searches. We determined the rotation axis by calculating the rotation needed to transpose the PDB model 3j77 (rotated) onto the PDB model 3j78 (non­rotated) using the script get_rot_axis.py provided in *Leopard-EM*. The vector between the center of the LSU and the SSU, which is necessary to adjust the defocus for each particle, was calculated using the script get_center_vector.py.

The constrained searches were then performed in four steps using the *constrained_search* program in *Leopard-EM*. By performing sequentially finer searches, we limit the number of cross-correlations, thus lowering the noise floor. First, a search was performed over the *Z* axis (ψ) in the range −13° to 2.5° in 1° steps. The second search, using the orientations from the first, was over the *Y* axis (θ) from −6.0° to 4.0° in 1° steps. The third search was over both angles between −5° and 5° in 0.5° steps. Finally, a search was performed over both angles between −0.5° and 0.5° in 0.1° steps. This final step included a defocus search in the range −100 and 100 Å in 20 Å steps. The results from the constrained searches were accumulated using the script sequential_threshold_processing.py, using a final threshold of one false positive for every 200 particles.

All scripts for the constrained search can be found at https://github.com/Lucaslab-Berkeley/Leopard-EM_manuscript.

### 2.7. 3D reconstructions

Although 3D reconstruction is not a native feature of *Leopard-EM*, external scripts were developed to extract all LSU particles with a detected SSU. The particle positions from the *Leopard-EM* results files were used to extract the particles in 512 × 512 pixel boxes, which were then normalized to have a mean of zero and a standard deviation of one. These extracted particles were saved as .mrcs files, with one file generated per micrograph. Subsequently, *RELION*-compatible STAR files were created using the Euler angles and CTF parameters in the *Leopard-EM* results. 3D volumes were generated for all particles, nonrotated particles and rotated particles using the *reconstruct* program in *RELION* (Scheres, 2012 ▸). After low-pass filtering the maps to 30 Å, masks for the full 80S ribosome were generated in *RELION*. These masks were manually modified in *ChimeraX* (Pettersen *et al.*, 2021 ▸) to create a mask for the SSU body. Postprocessing was performed in *RELION* with an automatically determined *B*-factor to generate the masked 3D maps and estimate the resolution. The maps were low-pass filtered to 8 Å for visualization purposes. The notebook detailing the procedure used to generate the 3D reconstructions is available at https://github.com/Lucaslab-Berkeley/Leopard-EM_manuscript/blob/main/figures/04f05d_reconstruct_untreated.ipynb.

### 2.8. Automated pixel-size search

Pixel-size optimization was performed on each of the 62 micrographs in the data set that had LSU detections, using the *Leopard-EM* program *optimize_template*, with the input positions and angles being generated from the LSU *refine_template* results. The simulation parameters were the same as those described in Section 2.4. The pixel size range was −0.05 to 0.05 Å in 0.01 Å increments for the coarse search and −0.008 to 0.008 Å in 0.001 Å increments for the fine search. The parameter write_individual_csv was set to true, meaning that the results file from every pixel-size run is exported for use in downstream processing. The results were then filtered to remove all particles with a *z*-score less than eight, and subsequently filtered to only select micrographs with more than 50 LSU detections, leaving 57 micrographs. The particles were sorted by *z*-score, and the mean of the 50 particles with the highest z-score was calculated.

The scripts for this can be found at https://github.com/Lucaslab-Berkeley/Leopard-EM_manuscript/blob/main/figures/03_pixel_size_plot.ipynb.

## 3. Results

### 3.1. *Leopard-EM* is an extensible, GPU-accelerated Python package for 2DTM

**Figure 1:**
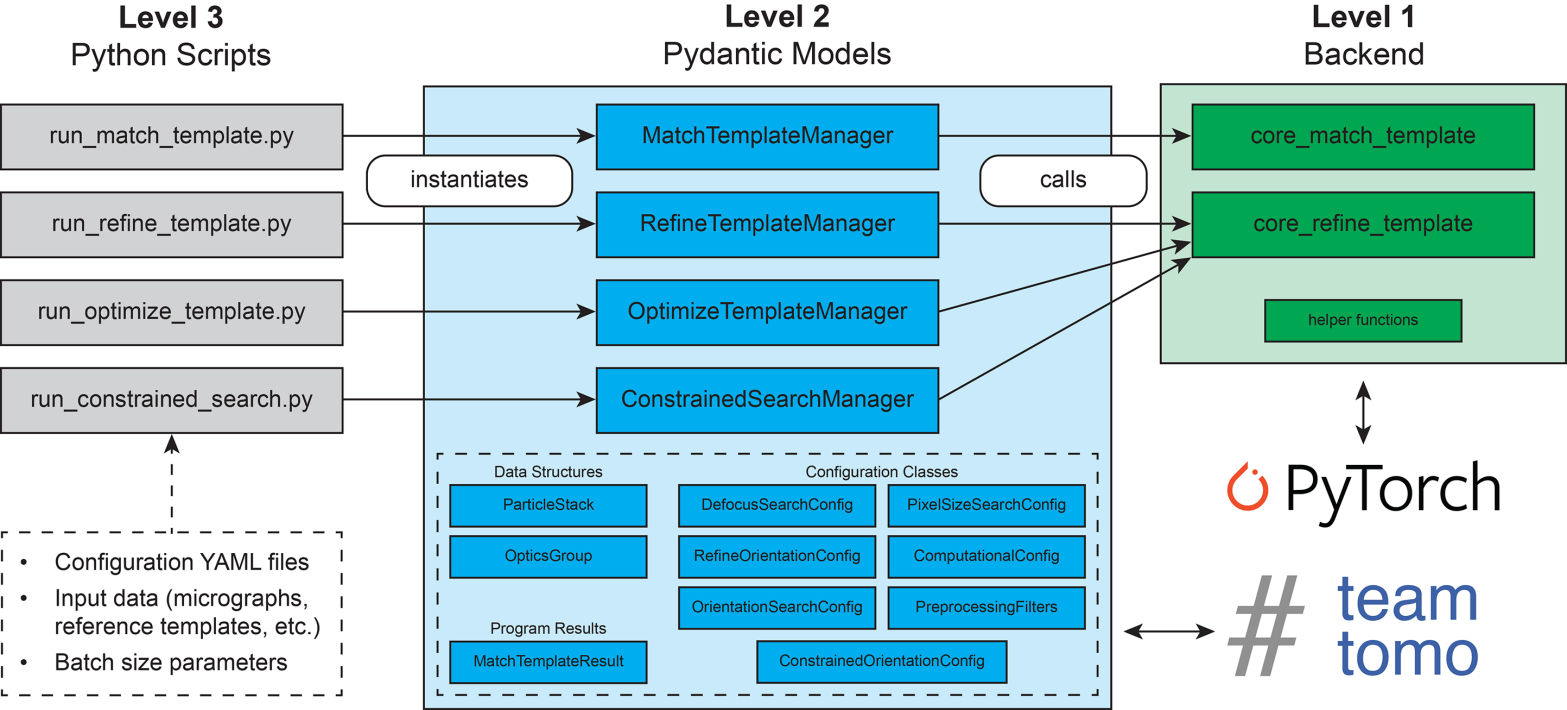
Overview of the multi-level *Leopard-EM* package. The third level of *Leopard-EM* comprises pre-written Python scripts (gray) for a particular program which instantiate a manager object at the second level (blue). Each manager object uses other Pydantic models, which encapsulate common data structures, define validation methods for inputs and act as connectors to cryo-EM data-processing methods defined in TeamTomo, to configure program inputs in a hierarchical manner. Manager objects call one of the backend functions in the first level (green) to run a program. The backend of the *Leopard-EM* package leverages PyTorch to interface with computer hardware, namely GPUs, to accelerate the computationally intensive stages of 2DTM.

To enable the adaptation of 2DTM to a wider range of biological problems, we have implemented the 2D template-matching algorithm (Rickgauer *et al.*, 2017 ▸, 2024 ▸; Lucas *et al.*, 2021 ▸) in a flexible and extensible Python package. Python has become the *lingua franca* for scientific programming and machine learning thanks to a rich infrastructure for data science and performant numerical libraries (Harris *et al.*, 2020 ▸; Pandas Development Team, 2020 ▸; Paszke *et al.*, 2019 ▸). Developing our 2DTM package within the Python ecosystem benefits from this pre-existing infrastructure and enables easy integration into larger cryo-EM workflows through Python scripts. We designed this 2DTM package with modularity in mind, separating user-facing programs, data-validation and preprocessing steps, and core backend functionality into three different levels (Fig. 1 ▸). We named this Python package *Leopard-EM*, for ***L**ocation and ori**e**ntati**o**n of **par**ticles found using two-**d**imensional t**E**mplate **M**atching*.

The core of the 2DTM method is the cross-correlation of two-dimensional projections from a reference model with a high-resolution cryo-EM micrograph (Lucas *et al.*, 2021 ▸). Calculating these cross-correlations greatly benefits from GPU hardware, and we implemented this core 2DTM functionality using PyTorch primitives for accelerated GPU computation. The computational backend of *Leopard-EM* is designed with a simple, data-oriented interface which expects an explicit definition of the search space, including a set of 3D orientations (for example, Euler angles) and a set of contrast transfer function (CTF) parameters used to search across different defocus planes. Each point in this search space corresponds to a specific combination of orientation and defocus which are used to generate a two-dimensional projection from a 3D reference template. By keeping the backend simple and separating it from the input parsing or preprocessing steps, we allow the possibility of using more performant backends other than PyTorch in the future without major modifications to the user-facing portions of the *Leopard-EM* package.

One layer up from the backend module, we included a set of custom classes built on Pydantic (Colvin *et al.*, 2025 ▸), a data-validation library for Python, to facilitate input configuration, data parsing and export of results in *Leopard-EM* (Fig. 1 ▸). These Python classes model input and output data structures, implement preprocessing steps, define the 2DTM search space and dispatch the computation to the backend module. By adopting this object-oriented design, we reduce the overall complexity by reusing common structures and methods as well as moving complicated, single-time preprocessing steps out of the backend. This modular framework will enable the rapid development of 2DTM workflows.

The final layer of *Leopard-EM* is the user-facing interface, which consists of easily configurable Python scripts for running *Leopard-EM* programs. Each program is configured using a YAML file, and the contents of the YAML file are mapped to *Leopard-EM* Pydantic models. Example YAML configurations and Python scripts can be found on the *Leopard-EM* GitHub page, and the configuration of each program is discussed in the online documentation (https://lucaslab-berkeley.github.io/Leopard-EM/).

Working closely with TeamTomo (https://teamtomo.org), a collaborative open-source project for cryo-EM infrastructure in Python, during the development of *Leopard-EM* has allowed us to focus on the 2DTM algorithm and reduce the time spent re-implementing common cryo-EM data-processing primitives. Specifically, we used the Fourier slice projection operation from *torch-fourier-slice* in the core 2DTM algorithm, and contributed packages to simulate electron scattering maps (*ttsim*3*d*), sample points in orientation space (*torch-so*3) and generate Fourier filters including the CTF (*torch-fourier-filter*). By making these primitive operations available as standalone Python packages, we hope to simplify future developments of Python-based cryo-EM workflows.

Importantly, developing *Leopard-EM* within Python, an interpreted language, has not incurred a performance penalty. Performance is comparable to a prior C++/CUDA implementation of 2DTM in the cryo-EM image-processing suite *cisTEM* (Lucas *et al.*, 2021 ▸; Grant *et al.*, 2018 ▸; marked with an asterisk in Table 1 ▸). Performance benchmarks on common GPU hardware are also listed in Table 1 ▸.

Adopting the modular framework as described above makes*Leopard-EM* easy to extend, enabling future enhancements to both the speed and sensitivity of 2DTM, as well as custom workflows to address specific biological questions.

### 3.2. *Leopard-EM* can locate and orient different macromolecules through exhaustive searches

In the standard 2DTM workflow, we first run the *Leopard-EM* program *match_template* which, analogously to the equivalent program in *cisTEM* (Lucas *et al.*, 2021 ▸), performs exhaustive orientation and defocus sampling to locate and orient particles within a micrograph. This program generates and cross-correlates reference template projections with the micrograph across all specified orientations and sample-defocus planes. The best-matching orientation and defocus, based on the highest cross-correlation value, is iteratively updated on a per-pixel basis as the program goes through the search space. After searching for all orientations and defoci, the maximum cross-correlation scores at each pixel are compared against a Gaussian noise model, and positive detections are assigned using a threshold chosen based on the expected number of false positives per micrograph (which is 1 by default; Rickgauer *et al.*, 2017 ▸). The orientation and defocus assignments are then further refined using the *Leopard-EM* program *refine_template*, which performs a local search around previously identified particles by sampling a finer grid of orientations and defocus values. The more precise results of *refine_template* can then be used for further downstream processing, such as visualizing the locations and orientations of identified molecules or 3D reconstruction.

**Figure 2:**
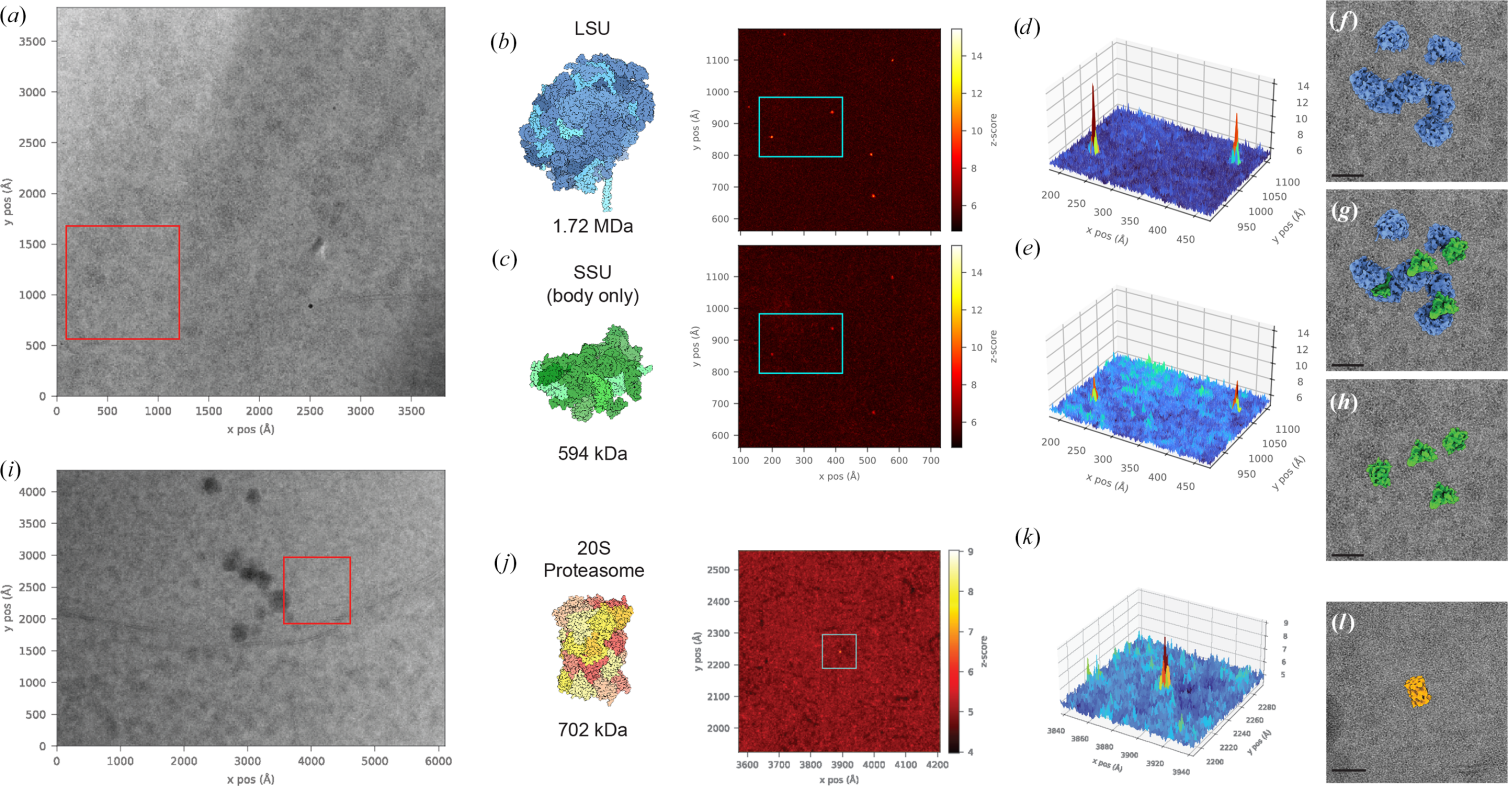
Representative two-dimensional template-matching (2DTM) results for searches run using a large ribosomal subunit (LSU), small ribosomal subunit body (SSU; modified from PDB entry 6q8y; Tesina *et al.*, 2019 ▸) and 20S proteasome (PDB entry 1ryp; Groll *et al.*, 1997 ▸) as reference templates. (*a*) Micrograph used for both LSU and SSU body 2DTM searches collected at 0.936 Å per pixel. (*b*) Left: LSU template with simulated molecular mass below. Right: 2DTM *z*-scores for LSU in the region of interest [red box in (*a*)]. (*c*) Left: SSU template with simulated molecular mass below. Right: 2DTM *z*-scores for SSU in the region of interest [red box in (*a*)]. (*d*, *e*) Enlarged *z*-score surface maps, output as scaled_mip from *Leopard-EM* within a cyan box for the LSU and SSU, respectively. (*f*, *g*, *h*) The relative position and orientations of LSU and SSU templates overlaid on the micrograph region of interest, where (*f*) shows the LSU alone, (*g*) shows both the LSU and SSU, and (*h*) shows the SSU alone. (*i*) Micrograph used for the 20S proteasome 2DTM search collected at 1.06 Å per pixel. (*j*) Left: proteasome template with simulated molecular mass below. Right: 2DTM *z*-scores for the proteasome search in the region of interest [red box in (*i*)]. (*k*) *Z*-score surface map for the identified proteasome peak. (*l*) 20S proteasome template at identified location and orientation overlaid on the region of interest. The scale bars in (*f*), (*g*), (*h*) and (*l*) correspond to 20 nm.

We confirmed that we can use the standard 2DTM workflow in *Leopard-EM* to detect single ribosomes in cryo-EM images of yeast lamellae, as previously demonstrated (Lucas *et al.*, 2021 ▸, 2022 ▸; Rickgauer *et al.*, 2024 ▸). We applied *Leopard-EM* to independently detect the large ribosomal subunit (LSU) and a fragment of the small ribosomal subunit (SSU) which lacked the head (Section 2.4) in cryo-EM images of yeast-cell cytoplasm (Figs. 2 ▸*a*–2 ▸*c*). Notably, the detected LSU and SSU particles align in space (Figs. 2 ▸*d* and 2 ▸*e*) and their orientations are consistent with the formation of 80S ribosomal complexes (Figs. 2 ▸*f*–2 ▸*h*), providing further validation of the significance of these detections.

Ribosomes are large, abundant and primarily consist of RNA, making them relatively prominent features in cellular cryo-EM images and, presumably, easier to detect with 2DTM. We show that we can also apply *Leopard-EM* to detect the protein-only 20S proteasome (Figs. 2 ▸*i*–2 ▸*l*). Applying *C*2 symmetry in the 2DTM search, we found 17 significant detections, excluding obvious membrane features, in eight of the 28 images searched. Although the total number of detections is less than the expected one false positive per image, examining the *z*-score for each identified particle allows us to estimate the false-positive probability of individual detections (Table 2 ▸). The observed high particle *z*-scores give confidence that many identified particles are unlikely to be false detections. This indicates that the naïve Gaussian noise approximation overestimates the false-positive rate in cellular images. Although many detections are likely to be missed, we found significant proteasome detections in the nucleus, close to the nuclear periphery (Fig. 2 ▸*i*), consistent with a previous report of enrichment at this cellular location in *Chlamyodomonas reinhardtii* (Albert *et al.*, 2017 ▸).

#### 3.2.1. Pixel-size refinement is essential for optimal detection of complexes with 2DTM

**Figure 3:**
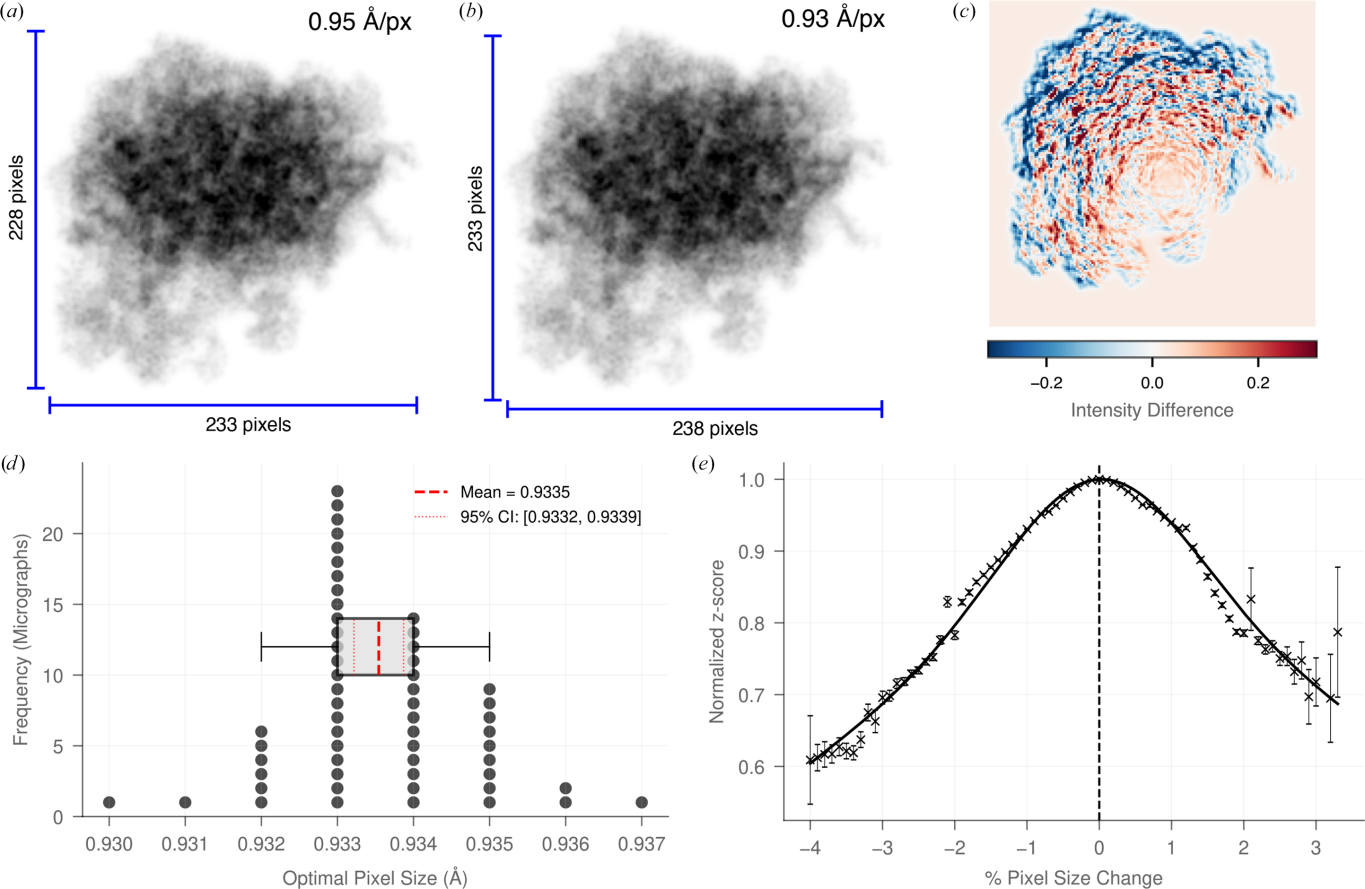
2DTM is sensitive to pixel-size errors of ∼0.2%. (*a*, *b*) Projection images from the same orientation of the LSU template with different pixel sizes: 0.95 Å (*a*) or 0.93 Å (*b*). An ∼2% difference causes significant changes in the size of template projections. (*c*) Intensity plot showing the difference in the intensity between (*a*) and (*b*), with each projection normalized to a mean of zero and a standard deviation of 1, showing a large change in intensity from 0.95 to 0.93 Å. (*d*) Histogram of the mean optimal pixel size in each micrograph with more than 50 LSU detections above a *z*-score of 8. The box plot shows the interquartile range and the whiskers represent the 5th and 95th percentiles. The mean and 95% confidence intervals of these pixel sizes are also plotted. 90% of the data were <0.2% from the mean. (*e*) A scatter plot showing the average reduction in *z*-score relative to the maximum for incorrect pixel sizes. The error bars are 95% confidence intervals. A sum of two Gaussian distributions is fitted to the data. The difference in pixel size shown in (*a*) and (*b*) causes a ∼20% decrease in 2DTM *z*-scores.

2DTM provides a readout of the agreement between a structural model and the image. For accurate detection, it is essential that the pixel sizes of both the model and the images are correct. Any discrepancy causes the template’s size and interatomic distances to differ from the target molecule (Fig. 3 ▸), reducing the cross-correlation score and lowering the 2DTM sensitivity. However, the extent of this effect has not been systematically evaluated.

To address this, we implemented a template pixel-size optimization step in *Leopard-EM* through the *optimize_template* program. This requires a pre-identified set of particles, which can be quickly obtained by running template matching on a cropped image. The optimization uses the *ttsim*3*d* package to simulate 3D volumes of the model at different pixel sizes, and then cross-correlates their projections from the annotated orientations with the image. The optimal pixel size is determined by maximizing the mean *z*-score of the top *N* peaks, where *N* is a user-definable parameter, or by maximizing the mean *z*-score for all peaks above a given *z*-score threshold. We note that this method simply scales the pixel size of the template to match the micrograph and is therefore agnostic to whether errors are in the model or micrograph pixel size. Therefore, it can only be used for microscope magnification calibration if the model pixel size is reliable, which is often true for X-ray-derived models but less certain for those built into cryo-EM maps.

To test the accuracy of this method, we used the 50 LSUs with the highest *z*-scores from each of 57 yeast lamella micrographs that contained more than 50 LSU detections with a *z*-score greater than 8. The optimal pixel size ranged from 0.930 to 0.937 Å per pixel, with a mean of 0.9333 ± 0.0003 Å per pixel, and 95% confidence intervals calculated using a Student’s *t*-test (Fig. 3 ▸*d*). 90% of the pixel sizes were within 0.2% of the mean.

To evaluate how pixel size affects the 2DTM *z*-score, we selected all particles from the 57 micrographs with *z*-scores greater than 8 and recalculated their *z*-scores across a range of deliberately incorrect pixel sizes. Each value was compared relative to the pixel size that yielded the highest mean *z*-score. The average across all particles was then calculated, and a sum of two Gaussians was used to fit the data (Fig. 3 ▸*e*). A pixel-size error of 0.5% results in a ∼2% drop in *z*-score. An error of 2% results in a ∼20% drop in *z*-score. Since SNR scales with the square root of molecular mass (Henderson, 1995 ▸; Rickgauer *et al.*, 2017 ▸), a 20% drop in *z*-score corresponds to a ∼45% increase in the minimum detectable molecular mass, making it significantly harder to identify small macromolecules. Considering that pixel-size errors exceeding 1% are frequent in cryo-EM data sets (Dickerson *et al.*, 2024 ▸), the pixel sizes of the model and image cannot be assumed to align. We therefore recommend performing pixel-size calibration for each combination of imaging conditions and template model, which can be performed using the *optimize_template* program in *Leopard-EM*.

### 3.3. A constrained search as an example of extensibility

The identification of macromolecules *in situ* using cryo-EM is fundamentally limited by the low-dose conditions required due to radiation damage. As a result, 2DTM is currently limited to detecting proteins with a molecular mass of at least 300 kDa *in situ* under ideal conditions (Rickgauer *et al.*, 2024 ▸). In 2DTM, the incorporation of high spatial frequency information creates narrow cross-correlation peaks with respect to orientation and defocus, which in turn requires fine angular and defocus sampling to reliably capture those peaks.

**Figure 4:**
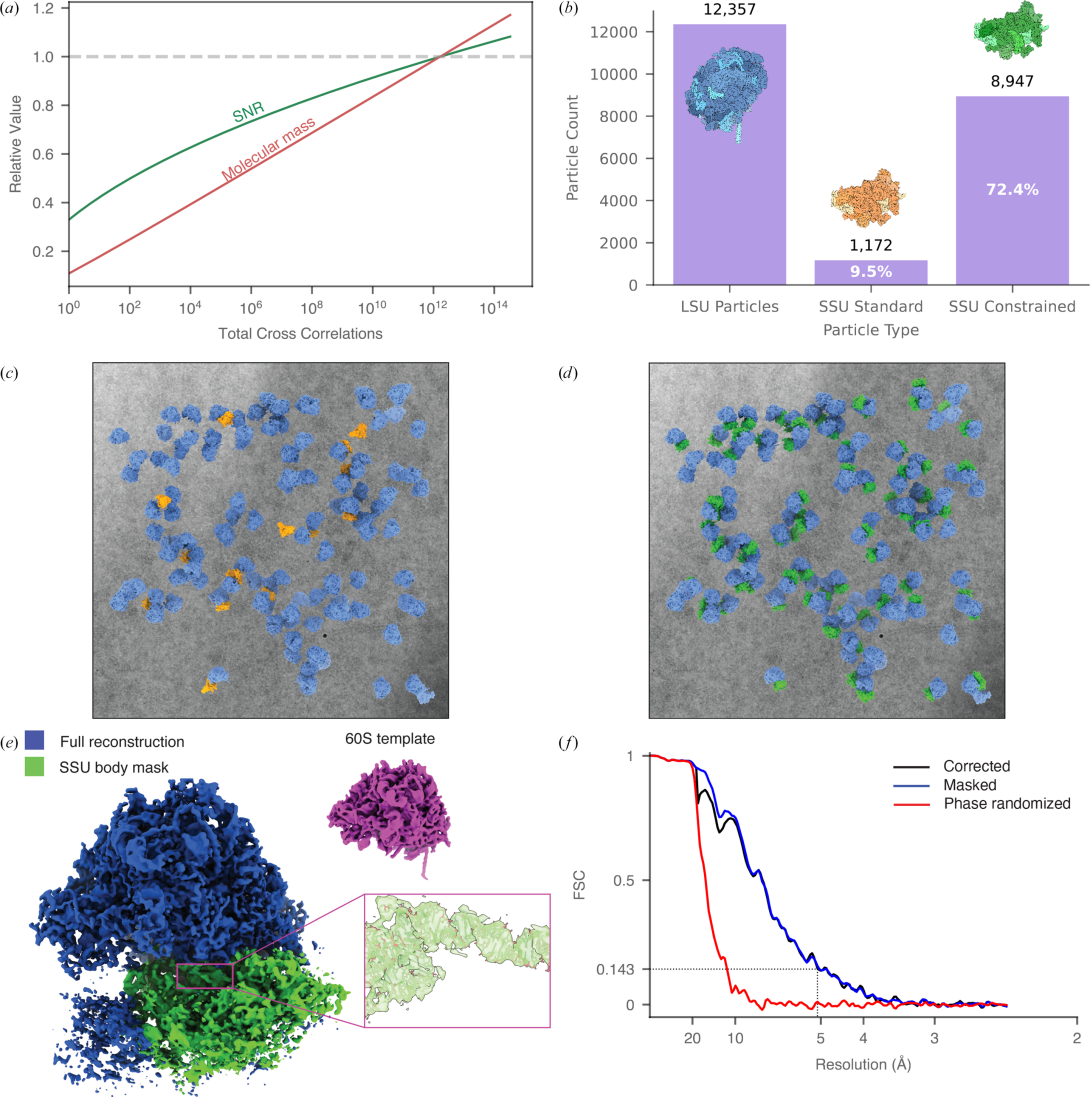
Constrained searches improve the sensitivity and functional resolution of 2DTM. Constraining the search space in 2DTM by limiting the number of cross-correlograms through known molecular positions and orientations decreases the signal-to-noise ratio (SNR) threshold for detection, thereby enabling the identification of smaller molecular complexes. (*a*) Plot showing the minimum detectable molecular mass at a false-positive rate of 1 in 200 particles, relative to the standard threshold of one false positive per micrograph. When both position and orientation are known, the detection threshold improves by approximately tenfold. (*b*) Plot showing the number of detections in 70 micrographs when performing an unconstrained search for the LSU and SSU body or constraining the search for the SSU body using positions and orientations from the LSU search (right). (*c*) Simulated slab of LSU particles (blue) and SSU particles (orange) found using the unconstrained 2DTM search overlaid on a representative micrograph. (*d*) The same LSU particles and micrograph now depicted with the constrained search SSU particles (green) showing that the constrained search increases 2DTM sensitivity. (*e*) Reconstructed volume of the LSU particles where a SSU particle had also been detected. The SSU is clearly present in the reconstruction despite not being present in the template. The PDB model 6q8y was docked into the map and a region of the SSU body (18S rRNA helix 44) is highlighted to demonstrate the fit between the map and the model. To estimate the resolution of the SSU body, a mask was created around the SSU body (green). This region was reconstructed to 5.1 Å resolution, as determined by a Fourier shell correlation of 0.143 (*f*) (Rosenthal & Henderson, 2003 ▸).

However, this fine sampling results in more independent comparisons and increases the chance of obtaining a high *z*-score purely by random chance, thus raising the effective noise floor and requiring a more stringent detection threshold. Reducing the number of cross-correlations directly reduces the probability of observing high *z*-scores due to noise alone, and therefore permits a lower detection threshold for the same false-positive rate (Rickgauer *et al.*, 2017 ▸). This can be achieved by restricting the search space in orientation (ϕ, θ, ψ) or position (*x*, *y*, *z*/defocus) (Fig. 4 ▸*a*). For example, if both the location and orientation of a particle are known in advance, the reduced number of comparisons can lower the detection threshold sufficiently to detect particles an order of magnitude smaller at a false-positive rate of 1 in 200 particles.

Beyond enabling the detection of smaller proteins, constrained searches also reduce false negatives when applied to larger molecules. We implemented this strategy in *Leopard-EM* by allowing users to restrict the search space via symmetry specifications or explicit angular ranges within the *match_template* program. Indeed, applying *C*2 symmetry when locating the proteasome allowed us to identify six additional significant detections by lowering the threshold for calling a significant detection compared with *C*1 symmetry (Table 2 ▸).

Additionally, we demonstrate the extensibility of *Leopard-EM* by developing a new program to exploit the positions and orientations of one molecule to increase the detectability of another molecule. This program, *constrained_search*, can serve as a model for others looking for customization within the 2DTM framework.

#### 3.3.1. Constrained searches identify small ribosomal subunits

We demonstrate this approach by searching for a fragment of the SSU lacking the dynamic head (which we hereafter refer to as the SSU body), which has a molecular mass in the template of approximately 594 kDa. An initial unconstrained search identified 1172 significant detections, <10% of the 12 357 significant detections identified when using the LSU as a template (Fig. 4 ▸*b*). Using the locations and orientations derived from the LSU 2DTM results, the constrained search yields significant SSU body detections adjacent to 72% of LSUs, an approximately eightfold increase (Fig. 4 ▸*b*). An example micrograph overlaid with these results reprojected is shown in Figs. 4 ▸(*c*) and 4 ▸(*d*). Although *Leopard-EM* does not natively perform 3D reconstruction, it is simple to convert the output files to a format compatible with other 3D reconstruction software, such as *RELION* (Scheres, 2012 ▸; Section 2.7). We used the 2DTM locations and orientations to reconstruct LSUs where we had detected an associated SSU body and recovered the density of the whole SSU, including the head, although it was not present in the template (Fig. 4 ▸*e*). The SSU reconstruction reached an estimated resolution of 5.1 Å (Fig. 4 ▸*f*), determined using a mask encompassing only the SSU body, which excluded the original LSU template to avoid template bias (Lucas *et al.*, 2023 ▸). The reconstructed density showed clear features consistent with this resolution, including discernible helical density consistent with helix 44 (Fig. 4 ▸*e*).

#### 3.3.2. Continuous distribution of small ribosomal subunit rotation and roll

**Figure 5:**
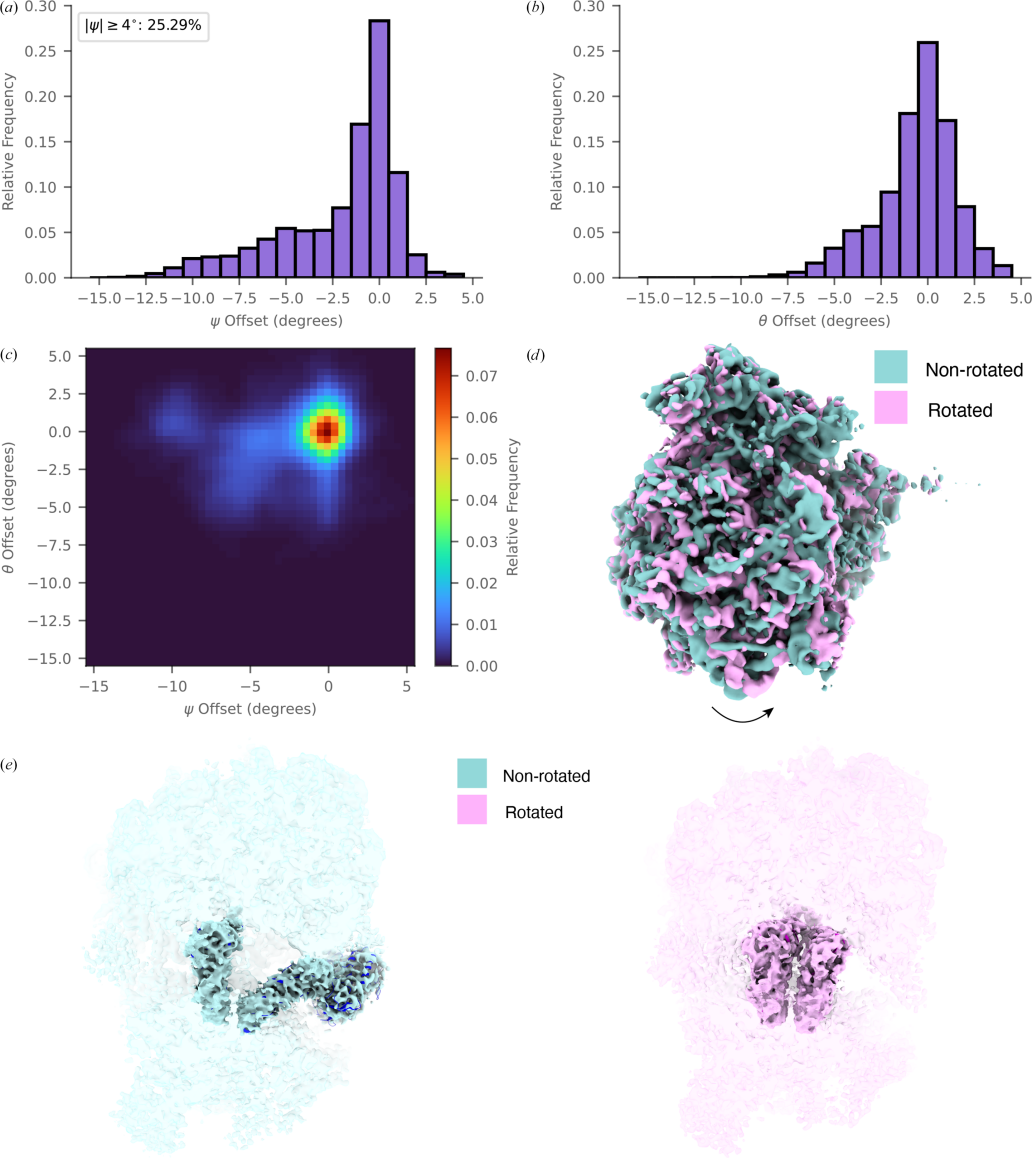
Constrained searches allow the identification of a continuous distribution of conformational states. (*a*) A histogram showing the angular distribution of SSU rotations relative to the LSU, as determined by constrained 2DTM. (*b*) A histogram showing the angular distribution of SSU at a secondary axis (subunit roll) relative to the LSU, as determined by constrained 2DTM. (*c*) A 2D heatmap combining the results shown in (*a*) and (*b*), where the intensity corresponds to the occupancy of SSUs. (*d*) Reconstructions of LSU particles classified by the output of 2DTM as having a rotated (≥4) or nonrotated (<4) SSU. (*e*) Reconstructions as in (*d*) where features within 5 Å of tRNAs and eEF1A (shown as ribbon diagrams) are highlighted as opaque by docking PDB entry 8z71 (Cheng *et al.*, 2025 ▸; blue) into the ‘nonrotated’ density (cyan) or PDB entry 8xu8 (Cheng *et al.*, 2025 ▸; pink) into the ‘rotated’ density (magenta).

The constrained search also enables us to capture continuous conformational heterogeneity at single-molecule resolution. The ribosome is a dynamic molecular machine comprising two subunits, an LSU and an SSU, which rotate relative to each other during mRNA translation and peptide synthesis (Korostelev, 2022 ▸). By comparing structural models determined in different rotational states, we constrain the search to one or two rotation axes, corresponding to intersubunit rotation (Ψ offset) and roll (θ offset) (Svidritskiy *et al.*, 2014 ▸). We find a predominate population with less than 2.5° Ψ offset relative to the nominally nonrotated ribosome template (Tesina *et al.*, 2019 ▸). The remaining approximately 25% of the ribosomes are in a rotated conformation (Fig. 5 ▸*a*), consistent with previous findings (Rickgauer *et al.*, 2024 ▸). We also find that there is a small offset on the orthogonal axis, consistent with SSU body roll (Fig. 5 ▸*b*), which coincides with the rotated state (Fig. 5 ▸*c*). To validate these results, we generated 3D reconstructions of LSUs classified as associated with an SSU body that is at least 4° rotated or less than 4° rotated, which could clearly be distinguished (Fig. 5 ▸*d*). Both reconstructions show density consistent with the SSU head and beak, which are not part of the SSU body template and therefore could not have been biased by the identification of rotation state. We additionally observed tRNAs (which were not included in either template) in distinct states in the two reconstructions. In the ‘nonrotated’ reconstruction, we observed clear density consistent with an accommodating tRNA in the A/T site with eEF1A and a tRNA in the P/P state (Fig. 5 ▸*e*), in agreement with previously observed states *in situ* (Cheng *et al.*, 2025 ▸). Consistent with our observation of a continuous distribution of rotation states, rather than a distinct population, the ‘rotated’ reconstruction is consistent with a mixed tRNA population. The strongest density showed features consistent with tRNAs in the A/A and P/P states (Fig. 5 ▸*e*), with additional density in the E-site consistent with a tRNA in the P/E state that was not observed in the ‘nonrotated’ reconstruction. The distinct tRNA conformations in the two reconstructions are consistent with classification based on the constrained search capturing different functional states.

In this experiment, a single SSU body template, taken from the structure of a nonrotated ribosome (Tesina *et al.*, 2019 ▸), was used in the constrained search to identify the rotation state. Within the SSU itself, there are conformational changes that accompany subunit rotation. We sought to minimize bias in the template by removing the SSU ‘head’, which undergoes a swivel relative to the SSU ‘body’. However, conformational differences within the SSU body template region are likely to remain, which could have biased the distribution of states and enriched for a subpopulation that more closely resembles the template. Therefore, the observed distribution of states cannot directly be inferred to represent a thermodynamic landscape. Future work to study a specific biological question could minimize this bias by combining the results from constrained searches using multiple templates representing different states, or by comparing relative changes in population distributions in different conditions. This example highlights the importance of template selection in interpreting the results from a template-based method such as 2DTM.

## 4. Conclusions and future perspectives

We have developed a new software package for 2DTM, *Leopard-EM*, which has been written with extensibility at the forefront. By implementing the package in Python and in a modular framework, we make this approach more accessible to the cryo-EM community and allow new features to be implemented more rapidly. This is crucial in a fast-moving field, where small improvements in sensitivity and speed can open up new areas of investigation. We demonstrate that the standard 2DTM workflow can be used in *Leopard-EM* to identify and align RNA-containing ribosomes and macromolecules without RNA. The main advantage of *Leopard-EM* lies in the ability to quickly implement nonstandard data-processing strategies, which we demonstrate by incorporating a constrained search and using it to increase sensitivity and identify continuous heterogeneity in SSU rotation states.

The sensitivity of 2DTM is currently limited to the detection of large complexes: greater than 300 kDa in the best-case scenario. This limitation is largely due to the difficulty in discriminating true positives from false positives. By constraining the search for the SSU using prior information from the LSU, we reduced the noise level, enabling us to distinguish true positives from false positives at lower SNR. Using this strategy, we recovered approximately eight times more SSU detections, demonstrating the potential of using prior information to improve the detection of smaller particles. This approach could easily be extended to incorporate other types of prior information, such as membrane features or other visible cellular structures.

We show that 2DTM can characterize molecular motions. During translation, the orientation of the SSU relative to the LSU changes. By performing a constrained local search for the SSU using two rotation axes defined on the LSU, we defined the distribution of rotation and roll states with single-molecule precision, without the need for 3D reconstruction and classification, consistent with Rickgauer *et al.* (2024 ▸).

The stringent threshold currently applied in 2DTM results in a high false-negative rate, meaning that only a subpopulation may be sampled. When conformation and compositional heterogeneity are assessed using strategies such as those described above, it is important to consider whether the observed distribution reflects the underlying distribution or may be biased by initial template selection.

The observation that the tRNAs in the ‘rotated’ reconstruction showed the strongest density in the classical state relative to the hybrid state (Fig. 5 ▸*e*), as would be expected for fully rotated ribosomes, is consistent with this population primarily consisting of ribosomes in intermediate rotation states, and relatively few fully rotated ribosomes. Finer classification along the rotation axis would likely reveal additional states and could be a way to investigate complex, multistep processes such as translation *in situ*. However, as described above, care in template selection is crucial.

*Leopard-EM* was designed to make it easy to develop custom workflows and new functionality. Structural cell biology is an emerging field and the role of untilted data collection and 2DTM are still being explored. Possible future improvements include substituting PyTorch for a custom, faster backend, the application of custom Fourier filters, the development of new scoring metrics and combining untilted with tilted data collection. Other modifications will likely emerge as this approach finds new applications.

The development of cryo-EM has always been dependent on software tools. *Leopard-EM* benefits from the modular tools that have been developed as part of TeamTomo that can be adapted to fit the question at hand. We expect that reducing the barrier to entry will enable a broader developer base to contribute to the development of new strategies to further improve single-molecule structural biology in cells.

## 5. Code and data availability

The code described in this manuscript can be found at the GitHub page for version 1.0: https://github.com/Lucaslab-Berkeley/Leopard-EM/tree/v1.0. The continuous development of the package can be followed at the main GitHub page: https://github.com/Lucaslab-Berkeley/Leopard-EM/.

Data used throughout this manuscript have been uploaded to EMPIAR-13107. Scripts for processing the data and generating the figures can be found on the Leopard-EM manuscript GitHub page at https://github.com/Lucaslab-Berkeley/Leopard-EM_manuscript.

## Figures and Tables

**Table 1:** Performance metrics for the *Leopard-EMmatch_template* program using common micrograph sizes across different GPUs The template volume was 512 × 512 × 512 voxels across all tests. Throughput is reported as the number of projections cross-correlated with the image per second. The full 2DTM search time is estimated using the given throughput for a standard search with 1.58 million orientations and 13 different defocus planes (20 598 240 total cross-correlations).An equivalent 2DTM search was run using the *cisTEMmatch_template* program (Lucas *et al.*, 2021 ▸; indicated by *) on the same computer hardware (AMD EPYC 9354 CPU, 256 GB memory, RTX 6000 Ada GPU). The comparable 2DTM search times indicate that developing *Leopard-EM* in Python, an interpreted language rather than a compiled language like C++, does not incur a performance penalty when leveraging numerical libraries such as PyTorch (Paszke *et al.*, 2019 ▸).

Program	GPU type	Image size	Throughput (cross-correlations s^−1^)	Time (h)
*Leopard-EM*	GeForce 2080 Ti	5760 × 4092 (K3)	217.1	26.40
*Leopard-EM*	GeForce 2080 Ti	4096 × 4096 (Falcon 4i)	343.0	16.70
*Leopard-EM*	RTX 6000 Ada/L40s	5760 × 4092 (K3)	431.7	13.30
Leopard-EM*	RTX 6000 Ada/L40s	4096 × 4096 (Falcon 4i)	744.5	7.69
*Leopard-EM*	RTX 6000 Blackwell Max-Q	5760 × 4092 (K3)	799.7	7.15
*Leopard-EM*	RTX 6000 Blackwell Max-Q	4096 × 4096 (Falcon 4i)	1394.7	4.10
*Leopard-EM*	A100	5760 × 4092 (K3)	530.2	10.79
*Leopard-EM*	A100	4096 × 4096 (Falcon 4i)	923.4	6.19
*Leopard-EM*	H100	5760 × 4092 (K3)	897.9	6.37
*Leopard-EM*	H100	4096 × 4096 (Falcon 4i)	1650.8	3.47
cisTEM*	RTX 6000 Ada/L40s	4096 × 4096 (Falcon 4i)	663.8	8.62

**Table 2:** Summary results for proteasome detections using 2DTM All particles which surpassed the *z*-score cutoff value for one false positive per micrograph and did not match membrane features are included. Particle *z*-scores were converted into false-positive probabilities using the Gaussian background noise model for determining 2DTM significance (Rickgauer *et al.*, 2017 ▸) for a 2DTM search using *C*2 symmetry. Detections listed below the empty row (particles 12–17) fall below the significance threshold for a 2DTM search using *C*1 symmetry, demonstrating that a smaller search space increases sensitivity.

Particle	Micrograph	Position *x* (pixels)	Position *y* (pixels)	*z*-score	False-positive probability
1	150_Mar12	3671	2113	9.033	0.000107
2	148_Mar12	1491	2042	8.732	0.000723
3	148_Mar12	1088	522	8.218	0.0186
4	148_Mar12	937	499	8.184	0.0230
5	148_Mar12	1662	777	8.137	0.0308
6	53_Mar11	550	1504	8.078	0.0443
7	146_Mar12	1377	1424	7.843	0.182
8	138_Mar12	5252	2680	7.840	0.185
9	25_Mar11	3611	2281	7.816	0.213
10	55_Mar11	4941	3230	7.803	0.228
11	146_Mar12	2227	2553	7.769	0.276

12	148_Mar12	580	2300	7.758	0.291
13	53_Mar11	3947	2742	7.746	0.311
14	138_Mar12	4140	484	7.717	0.361
15	146_Mar12	91	1931	7.695	0.402
16	149_Mar12	4270	326	7.637	0.524
17	149_Mar12	742	1944	7.625	0.551
